# Hormonal status in protracted critical illness and in-hospital mortality

**DOI:** 10.1186/cc10010

**Published:** 2011-02-03

**Authors:** Tarek Sharshar, Sylvie Bastuji-Garin, Andrea Polito, Bernard De Jonghe, Robert D Stevens, Virginie Maxime, Pablo Rodriguez, Charles Cerf, Hervé Outin, Philippe Touraine, Kathleen Laborde

**Affiliations:** 1 Department of Intensive Care Medicine, AP-HP, Raymond Poincaré Hospital, University Versailles Saint-Quentin en Yvelines, 104 bd Raymond Poincaré, Garches F-92380, France; 2 Department of Clinical Research and Public Health, AP-HP, Henri Mondor Hospital, Université Paris Est Créteil (UPEC), Faculty of Medicine, 51 avenue du Maréchal de Lattre de Tassigny, Créteil F-94010, France; 3 Department of Intensive Care Medicine, Poissy-Saint-Germain en Laye Hospital, 10 rue du champ gaillard, Poissy F-78300, France; 4 Departments of Anesthesiology and Critical Care Medicine; Neurology; and Neurosurgery, Johns Hopkins University School of Medicine, 600 North Wolfe Street, Baltimore, MD 21287, USA; 5 Department of Medical Intensive Care Medicine, AP-HP, Henri Mondor Hospital, Université Paris Est Créteil (UPEC), Faculty of Medicine, 51 avenue du Maréchal de Lattre de Tassigny, Créteil, F-94010, France; 6 Department of Surgical Intensive Care Medicine, AP-HP, Henri Mondor Hospital, Université Paris Est Créteil (UPEC), Faculty of Medicine, 51 avenue du Maréchal de Lattre de Tassigny, Créteil, F-94010, France; 7 Department of Endocrinology and Reproductive Medicine, AP-HP, Pitié-Salpêtrière Hospital, Pierre Marie Curie University, 47-83, boulevard de l'Hôpital, Paris F-75013, France; 8 Department of Physiology AP-HP, Necker Enfants-Malades Hospital, University Paris Descartes, 149, rue de Sèvres, Paris, F-75743, France

## Abstract

**Introduction:**

The aim of this study was to determine the relationship between hormonal status and mortality in patients with protracted critical illness.

**Methods:**

We conducted a prospective observational study in four medical and surgical intensive care units (ICUs). ICU patients who regained consciousness after 7 days of mechanical ventilation were included. Plasma levels of insulin-like growth factor 1 (IGF-1), prolactin, thyroid-stimulating hormone, follicle-stimulating hormone, luteinizing hormone, estradiol, progesterone, testosterone, dehydroepiandrosterone (DHEA), dehydroepiandrosterone sulfate (DHEAS) and cortisol were measured on the first day patients were awake and cooperative (day 1). Mean blood glucose from admission to day 1 was calculated.

**Results:**

We studied 102 patients: 65 men and 37 women (29 of the women were postmenopausal). Twenty-four patients (24%) died in the hospital. The IGF-1 levels were higher and the cortisol levels were lower in survivors. Mean blood glucose was lower in women who survived, and DHEA and DHEAS were higher in men who survived.

**Conclusions:**

These results suggest that, on the basis of sex, some endocrine or metabolic markers measured in the postacute phase of critical illness might have a prognostic value.

## Introduction

Critical illness is associated with various endocrinological dysfunctions, which has also been linked to increased mortality, but this association has been reported primarily in acute rather than protracted (>7 days) critical illness [1-4]. As endocrine status changes with the course of critical illness [5], the prognostic value of a given hormone may differ between the acute and prolonged phases. There is an extensive literature on the prognostic value of endocrinological markers in the acute phase of critical illness, in contrast to the prolonged phase. Most hormonal studies on protracted critical illness have either included a small or particular cohort [6] or assessed one endocrine axis [7]. Therefore, we assessed the relationships between various endocrine markers and in-hospital mortality in a large population of patients with protracted critical illness [8]. The endocrine functions that we have assessed included the adrenal, thyrotropic, somatotropic and gonadotropic axes, as they have been shown to be impaired during and after critical illness [1-4] and play a major role not only in the response to stress [9,10] but also with regard to patient outcomes [2,3]. These endocrine markers were assessed in a study on ICU-acquired paresis [11] because they affect muscle metabolism. However, although the present study is based on the same population [8,12] and the same hormonal measurements [11] as previously published ones, its objective (that is, in-hospital mortality) is entirely original.

## Materials and methods

### Patients

Briefly, the study was conducted prospectively between June 2003 and June 2005 in four ICUs (two medical, one surgical and one medicosurgical). Patients who required at least mechanical ventilation were screened daily for awakening and comprehension using five simple verbal commands as previously described [13,14]. Patients were enrolled in the study and hormonal assays were performed on the first day when awakening and comprehension were satisfactory (day 1). Therefore, patients without successful awakening were not included. The study protocol was approved by the Ethics Committee of Saint-Germain-en-Laye, France. Informed consent was obtained from all patients.

Demographic characteristics, category of admission, comorbidities and intensive care unit (ICU) admission diagnosis were recorded, as well as the severity of critical illness determined using the Simplified Acute Physiology Score II (SAPS II) [15] and the Organ Dysfunctions and/or Infection score [16]. The mean blood glucose levels and the cumulative dose of corticosteroids (expressed as hydrocortisone-equivalent dosage) between ICU admission and inclusion in the study were calculated for each patient.

### Endocrinological measurements

Plasma follicle-stimulating hormone (FSH), lutenizing hormone (LH) and prolactin concentrations were measured using radioimmunometric assays (RIAs) (Access 2; Beckman Coulter, Villepinte, France) as described elsewhere [11]. Plasma concentrations of testosterone, estradiol and dehydroepiandrosterone (DHEA) were determined by performing RIA after ether extraction. Plasma concentrations of dehydroepiandrosterone sulfate (DHEAS), progesterone, cortisol and insulin-like growth factor 1 (IGF-1) were measured directly by RIA (CIS Bio International, Gif-sur-Yvette, France). Plasma cortisol levels measured in patients still being treated with hydrocortisone were not taken into account in the analysis. Plasma concentrations of thyroid-stimulating hormone were determined by using a third-generation sandwich immunoassay (Immunotech Beckman Coulter, Villepinte, France). Plasma levels were considered abnormally low when they were below the lowest normal value.

In men, independently of age, hypogonadism was considered when plasma testosterone levels were below 3 ng/ml [17]. Hypogonadism was considered secondary (SH) when FSH and LH concentrations were below 5 mU/l and primary (PH) when FSH and LH levels were above 10 mIU/l [17].

Women were considered postmenopausal if they were older than 55 years of age or if they reported amenorrhea for 1 year or more. Because of the small number of premenopausal women (*n *= 8), sex-dependent hormones were analyzed only in postmenopausal women. In postmenopausal women, PH was considered to be the rule. Hypogonadism was considered SH when LH and FSH levels were inappropriately low (<10 mIU/l) in the presence of a low estradiol level (<10 pg/ml) in postmenopausal women [17].

### Mortality

The end point of the study was in-hospital mortality. Therefore, we assessed the association of in-hospital mortality with day 1 plasma levels of nongonadic hormones measured in the whole population and of gonadic hormones separately for postmenopausal women and men. For each nonsurvivor, the cause of death was determined by two independent observers on the basis of a review of the medical notes and charts, and deaths were classified as being due to sepsis or not. Deaths attributed to infection were those in which an infection-related complication developed after initial awakening, including septic shock, multiple organ failure, acute respiratory distress syndrome and hypoxemic pneumonia. Cases in which death was associated with a decision to limit or withdraw care were also recorded [18].

### Statistical analyses

Variables were recorded upon admission, between admission and awakening, at awakening (hormonal data) and at discharge (Tables 1 and 2). Continuous variables were not dichotomized and were reported as medians with interquartile ranges, and categorical variables were coded as 1 or 0 and reported as percentages. The Mann-Whitney *U *test was used for comparison of continuous variables, and the χ^2 ^or Fisher's exact test was used to assess categorical variables.

**Table 1:** Patients' clinical characteristics and outcomes^a^

Patient demographics, *N *= 102 (100%)	Data
Median age, yr (IQR)	66 (51 to 78)
COPD^b^, *n *(%)	39 (38%)
Chronic cardiac insufficiency^b^, *n *(%)	28 (27.5%)
Medical admission, *n *(%)	71 (69.6%)
Median SAPS II at ICU admission (IQR)	46 (38 to 55)
	
From admission to awakening (day 1)	
Septic shock^c^, *n *(%)	53 (52%)
Median days with failure of ≥2 organs^d^, days (IQR)	8 (7 to 11)
Median duration of mechanical ventilation, days (IQR)	10.0 (8.0 to 14.0)
Mean blood glucose, mM/l (IQR)	7.6 (6.9 to 8.8)
Use of vasopressors, *n *(%)	77 (75%)
Use of corticosteroids, *n *(%)	64 (63%)
Median corticosteroid dose, 10^3 ^g (IQR)	1.0 (0 to 1.9)
Median delay from steroid administration to day 1, days (IQR)	3.0 (1.0 to 8.0)
Use of NMBA, *n *(%)	40 (39%)
	
At awakening (day 1, *n *= 86)	
Median SAPS II (IQR)	30 (23 to 26)
	
After awakening	
Median ICU length of stay, days (IQR)	23 (15 to 35)
ICU mortality, *n *(%)	15 (15%)
In-hospital mortality, *n *(%)	24 (24%)

^a^IQR, interquartile range; COPD, chronic obstructive pulmonary disease; ICU, intensive care unit; SAPS II, Simplified Acute Physiology Score II [15]; NMBA, neuromuscular blocking agent; ^b^diagnosis of COPD and chronic cardiac insufficiency were based on clinical history; ^c^septic shock was defined as the administration of catecholamines and a concomitant documented infection after exclusion of other causes of shock; ^d^renal, hepatic, and hematological failure were defined according to the Organ Dysfunctions and/or Infection score [16].

**Table 2:** Comparison between hospital survivors and nonsurvivors^a^

Patient demographics, *N *= 102 (100%)	Hospital survivors (*n *= 78)	Hospital nonsurvivors (*n *= 24)	***P *value**^**b**^	**OR (95% CI)**^**c**^
Women, n (%)	24 (30.8)	13 (54.2)	0.05	2.7 (0.97 to 6.8)
Median age, yr (IQR)	62 (47 to 77)	69 (58 to 80)	0.13	
From admission to awakening (day 1)				
Mean blood glucose^d^, mM/l (IQR)	7.6 (6.8 to 8.6)	8.3 (7.2 to 9.7)	0.07	1.2 (0.95 to 1.4)
Women	8.0 (7.0 to 8.7)	9.4 (7.8 to 11.1)	0.03	1.5 (0.98 to 2.2)
Men	7.4 (6.7 to 8.6)	7.2 (6.7 to 8.7)	0.89	
At awakening (day 1)				
Median SAPS II (IQR)	28 (21 to 34)	35 (29 to 41)	0.007	1.1 (1.0 to 1.1)
Median FSH^e^, mIU/ml (IQR)				
Women	2.9 (0.75 to 17.9)	1.6 (0.68 to 4.7)	0.41	
Men	3.9 (1.9 to 7.6)	3.8 (1.6 to 6.5)	0.93	
Median LH^e^, mIU/ml (IQR)				
Women	0.35 (0.2 to 3.0)	0.21 (0.21 to 1.2)	0.61	
Men	4.35 (2.2 to 6.5)	6.9 (0.63 to 13)	0.56	
Median prolactin, ng/ml (IQR)	9.5 (5.2 to 16)	8.3 (5.1 to 15)	0.54	
Median estradiol^e^, pg/ml (IQR)				
Women	10 (10 to 28)	10 (10 to 12)	0.82	
Men	14.5 (10 to 23)	10 (10 to 24)	0.79	
Median testosterone^e^, ng/ml (IQR)				
Women	0.09 (0.07 to 0.18)	0.07 (0.07 to 0.16)	0.81	
Men	0.78 (0.35 to 1.70)	0.63 (0.43 to 1.1)	0.57	
Median cortisol^f^, ng/ml (IQR)	16.0 (12.0 to 23.0)	23.0 (18.5 to 34.5)	0.01	4.3 (1.5 to 12.1)
Women	15.5 (12.0 to 25.0)	23.0 (20.0 to 25.0)		
Men	16.0 (12.0 to 23.0)	22.0 (14.0 to 41.5)		
Median DHEA^e^, ng/ml (IQR)				
Women	0.30 (0.30 to 0.66)	0.30 (0.30 to 0.89)	0.50	
Men	0.59 (0.30 to 1.80)	0.30 (0.30 to 0.45)	0.01	0.2 (0.04 to 0.97)
Median DHEAS^e^, ng/ml (IQR)				
Women	262 (107 to 469)	366 (79 to 580)	0.86	
Men	486 (184 to 1,141)	198 (100 to 310)	0.04	0.2 (0.03 to 0.8)
Median progesterone, ng/ml (IQR)				
Women	0.23 (0.05 to 0.29)	0.24 (0.04 to 0.69)	0.49	
Median SH (%)	57 (73%)	18 (75%)	1.00	
Median PH (%)				
Men	19 (35%)	6 (55%)	0.31	
Median TSH, mIU/ml (IQR)	1.25 (0.52 to 2.35)	1.34 (0.74 to 2.12)	0.68	
Median IGF-1, ng/ml (IQR)	78 (56 to 112)	65 (46 to 70)	0.007	0.2 (0.07 to 0.6)
Women	73.5 (50.5 to 113.5)	59.5 (57.5 to 69.0)		
Men	81 (59 to 111)	65.0 (50.0 to 73.0)		

^a^SAPS, Simplified Acute Physiology Score II [15]; DHEA, dehydroepiandrosterone; DHEAS, dehydroepiandrosterone sulfate; FSH, follicle-stimulating hormone; LH, luteinizing hormone; TSH, thyroid-stimulating hormone; IGF-1, insulin-like growth factor 1; SH secondary hypogonadism; PH, primary hypogonadism; IQR, interquartile range; OR, odds ratio; 95% CI, 95% confidence interval; ^b^*P *values were derived from performing the Mann-Whitney *U *test or Fisher's exact test as appropriate; ^c^OR and 95% CI were estimated by using exact logistic regression models; ^d^ORs estimated after dichotomization on median value; ^e^assessed in 85 patients, including 56 men and 29 postmenopausal women, among whom 11 men and 11 women died in the hospital, respectively; ^f^plasma cortisol levels of 83 patients were taken into account in the analysis; the other 19 patients were still being treated with hydrocortisone at the time the blood sample was taken and thus were excluded from the cortisol measurement.

Survivors and nonsurvivors were compared on *a priori *selected variables, including sex, age, SAPS II and hormonal measurements (Table 2). Odds ratios (ORs) and 95% confidence intervals (95% CIs) were estimated by using exact logistic regression models for variables associated with survival with *P *< 0.05. ORs were stratified by sex for gonadic hormones. Because of the low number of events, multivariate analysis including variables associated with in-hospital mortality could not be performed.

*P* ≤ 0.05 was considered statistically significant. All significance tests were two-tailed. Data were analyzed using the Stata release 8.0 software (StataCorp. 2003, College Station, TX, USA) and the StatXact and LogXact software programs (Cytel Inc., Cambridge, Massachussets, USA).

## Results

### Patients' characteristics

The study patients' characteristics are presented in Table 1. Twenty-four patients (24%) died in the hospital, including 15 in the ICU. Fourteen patients (58%) died as a result of an infection-related complication that developed after initial awakening. Six patients (25%) died as a result of severe chronic cardiac or respiratory insufficiency, three patients (13%) died as a result of sudden death and one patient (4%) died as a result of generalized cancer. A decision to limit or withdraw life-sustaining measures was made in 11 patients.

In the overall population, in-hospital mortality was significantly associated with female sex and a higher day 1 SAPS II (Table 2). In-hospital nonsurvivors had significantly higher plasma cortisol levels and lower plasma IGF-1 levels than in-hospital survivors. Mean blood glucose levels between admission and awakening tended to be greater in in-hospital nonsurvivors.

Mean blood glucose levels were significantly higher in women who were nonsurvivors. Other plasma hormone levels, as well as the prevalence of SH, did not differ at a statistically significant level between the two groups.

In men, plasma levels of DHEA and DHEAS were significantly lower in nonsurvivors. The proportion of men with SH and PH, as well as plasma levels of gonadic hormones and mean blood glucose, did not show a statistically significant difference between the two groups.

## Discussion

Protracted critical illness is associated with dysfunction of the neuroendocrine axes and the adrenal gland [5,6,17,19-21], which is characterized by low circulating levels of hypophyseal and adrenal hormones, notably DHEA and DHEAS. In accord with our previous study [11], the present results are consistent with this endocrine pattern, indicating that hormonal status has been assessed at the postacute phase of critical illness.

We found that nonsurvivors had increased plasma cortisol levels, suggesting persisting stress. Increased plasma cortisol level was associated with decreased plasma DHEA and DHEAS levels in men who subsequently died, suggesting adrenal exhaustion [22]. Although the association of mortality with adrenal exhaustion has also been reported previously in septic shock [1,2], we do not have any explanation for the fact that it was observed only in men in the present study. Interestingly, neither high circulating cortisol levels nor adrenal exhaustion were related to the administration of corticosteroids, suggesting that corticosteroid therapy has no deleterious effect on adrenal function. This is an important finding, considering the controversy regarding the usefulness of corticosteroids in patients in septic shock [23]. Arlt *et al*. [1] previously showed a lack of association between DHEA (increased) and DHEAS (decreased) and that mortality was associated with an increased cortisol-to-DHEA ratio. However, these results were obtained when patients were in an early stage of septic shock. Conversely, Marx *et al*. [2] measured the plasma levels of adrenocortical hormones in 30 patients at the onset, the halfway point and the last day of sepsis, with a total duration of about 9 days. On the last day of sepsis, they found that plasma levels of cortisol and DHEA tended to be higher and those of DHEAS were lower in nonsurvivors. The discrepancy between the DHEA findings between the study by Marx *et al*. and our study might result from differences in the populations studied, especially with regard to admission diagnosis (sepsis vs. critical illness) and male-to-female sex ratio. The immune system-activating properties of DHEA may account for the association of DHEA levels with mortality [1,2]. These findings would support an assessment of the benefit of DHEA treatment in the postacute phase of critical illness, notably in men [24].

We found that in-hospital mortality was associated with low plasma IGF-1 levels. To our knowledge, this postacute phase relationship has been assessed in only one small cohort study [6]. A low IGF-1 level is considered a valuable marker of growth hormone (GH) deficiency, which is considered deleterious [25] and has inspired clinical trials [26,27]. Unfortunately, one randomized clinical trial has shown that the administration of GH increased mortality in critically ill patients [26]. Because GH was administered during the acute phase of critical illness in the Takala *et al*. trial [26], one may argue that GH administration should be tested during the prolonged phase of critical illness. Moreover, it has recently been shown that critical illness-associated mortality was not associated with IGF-1 level but with increased GH level (measured in the acute phase) [28]. It has to be noted that decreases in circulating IGF-1 levels can result from various causes frequently encountered in critically ill patients, such as malnutrition, chronic liver disease or diabetes [17]. In contrast to previous reports [29,30], we did not find that plasma IGF-1 levels differed between women and men.

Female sex and increased blood glucose levels have been shown to be independently associated with increased mortality [31-33]. Therefore, these relationships can support our finding that blood glucose levels were higher in women who did not survive. It is also known that menopause is associated with type 2 diabetes mellitus. Preexisting diabetes was not more frequent in female patients who did not survive. It is conceivable that the conjunction of menopause and critical illness induce insulin resistance. Although such a benefit has not been reported in a large trial [34,35], it would be worth assessing the effect of strict glucose control in postmenopausal female patients in the ICU.

### Limitations of the study

The biological effects of hormones depend not only on their circulating levels but also on specific and nonspecific hormone-binding proteins and on the expression and regulation of hormone receptors. Since we did not assess binding protein levels or hormone receptor activity, we cannot exclude that a given hormone is associated with mortality on the basis of serum levels alone. Similarly, tissue hormone levels might also have a prognostic value, but obviously they are not assessable in a living patient. Thus, Arem *et al*. [36] found that tissue thyroid hormone levels were lower in most organs of more patients who died as a result of critical illness than in those of patients who died as a result of trauma. Finally, single circulating levels of hormones must be interpreted with caution because these levels may fluctuate with time, and dynamic assessments were not performed in the present study [17]. Similarly, assessment of pulsatile secretion of hypothalamohypophyseal hormones would also have been interesting. Because such assessments require repeated measurements, comprehensive hormonal studies have included a relatively small number of patients [37].

We acknowledge that a statistical association does not signify a causal relationship. Endocrinological dysfunction and mortality might be two independent consequences of critical illness. Because of the relatively low number of events, we did not perform multivariate analyses to determine whether endocrinological dysfunction was independently associated with in-hospital mortality. It is also possible that a larger patient cohort would have allowed us to identify other endocrinological factors. Despite these limitations, our study remains original, as we have assessed the relationships between various hormones and mortality at the postacute phase of critical illness in a patient cohort that is relatively large in comparison with other similar studies. It has to be noted that hormones were not chosen at random, but rather because they might affect outcomes, including even gonadotropic hormones [3,38].

We have used the term "protracted" because assessment of plasma hormone levels was done after the seventh day of critical illness. Indeed, this time point is often used to discriminate the acute phase from the postacute phase of critical illness. We acknowledge that this definition is too simple, because "time" is not the same for all patients and all types of critical illness. From a clinical point of view, awakening is a major milestone in the course of critical illness. It often indicates recovery, and it is a time when important therapeutic decisions are made, such ventilator weaning or physiotherapy.

## Conclusions

We found that in-hospital mortality was associated with high plasma cortisol and low plasma IGF-1 levels in the whole patient population, with low plasma DHEA and DHEAS levels in men and with increased blood glucose levels in women. Before attempting to conduct a clinical trial on hormonal therapy, we think that these associations should be confirmed in a larger patient cohort and that their pathogenic mechanisms should be elucidated.

## Key messages

• The impact of endocrinological dysfunction in the postacute phase of critical illness has been scantly assessed.

• The adrenal, thyrotropic, somatotropic and gonadotropic axes were assessed in 102 patients (65 men and 37 women) who had required mechanical ventilation for at least seven days (median, 10 days).

• The in-hospital mortality rate was 24%.

• The plasma level of IGF-1 was higher and that of cortisol was lower in survivors, regardless of sex.

• Plasma levels of DHEA and DHEAS were higher in men who survived.

## Abbreviations

DHEA: dehydroepiandrosterone; DHEAS: dehydroepiandrosterone sulfate; FSH: follicle-stimulating hormone; ICU: intensive care unit; IGF-1: insulin-like growth factor 1; IQR: interquartile range; LH: luteinizing hormone; ODIN: Organ Dysfunctions and/or Infection score; PH: primary hypogonadism; SAPS II: Simplified Acute Physiology Score II; SH: secondary hypogonadism; TSH: thyroid-stimulating hormone.

## Competing interests

The authors declare that they have no competing interests

## Authors' contributions

TS conceived of the study, helped recruit the patients and wrote the manuscript. SBG participated in the design of the study, performed the statistical analysis and helped to draft the manuscript. AP helped to draft the manuscript. BDJ participated in the design of the study and helped to recruit the patients and draft the manuscript. RDS helped to draft the manuscript. VM helped to recruit the patients and draft the manuscript. PR helped to recruit the patients. CC helped to recruit the patients. HO helped to recruit the patients. PT participated in the design of the study and helped to draft the manuscript. KL participated in the design of the study, performed the measurement of plasma hormones levels and helped to draft the manuscript.
